# The effect of a cluster-randomized controlled trial on lifestyle behaviors among families at risk for developing type 2 diabetes across Europe: the Feel4Diabetes-study

**DOI:** 10.1186/s12966-021-01153-4

**Published:** 2021-07-01

**Authors:** Vicky Van Stappen, Greet Cardon, Marieke De Craemer, Christina Mavrogianni, Nataliya Usheva, Jemina Kivelä, Katja Wikström, Pilar De Miquel-Etayo, Esther M. González-Gil, Anett S. Radó, Anna Nánási, Violeta Iotova, Yannis Manios, Ruben Brondeel

**Affiliations:** 1 Department of Movement and Sports Sciences, Ghent University, Ghent, Belgium; 2 Research Foundation Flanders (FWO), Egmontstraat 5, 1000 Brussels, Belgium; 3 Department of Rehabilitation Sciences, Ghent University, Ghent, Belgium; 4 Department of Nutrition and Dietetics, Harokopio University, School of Health Science & Education, Athens, Greece; 5 Clinic of Paediatric Endocrinology, Medical University Varna, Varna, Bulgaria; 6 Finnish Institute for Health and Welfare, Helsinki, Finland; 7 GENUD (Growth, Exercise, Nutrition and Development), University of Zaragoza, Zaragoza, Spain; 8 Department of Biochemistry and Molecular Biology II, Center of Biomedical Research (CIBM), Instituto de Nutrición y Tecnología de los Alimentos, Universidad de Granada, Granada, Spain; 9 CIBER Fisiopatología de la Obesidad y Nutrición, Instituto de Salud Carlos III, Madrid, Spain; 10 Debreceni Egyetem (UoD), University of Debrecen, Debrecen, Hungary

**Keywords:** Physical activity, Sedentary behavior, Eating behavior, Family, Lifestyle intervention, Prevention, Type 2 diabetes

## Abstract

**Background:**

This study investigated the effect of the Feel4Diabetes-intervention, a 2-year multilevel intervention, on energy balance-related behaviors among European families at risk for developing type 2 diabetes. Intervention effects on self-reported physical activity, sedentary behavior and eating behaviors were investigated across and within the participating countries: Belgium, Finland, Greece, Spain, Hungary and Bulgaria.

**Methods:**

Families were recruited through schools, located in low socio-economic status areas. In total, 4484 families at risk for developing type 2 diabetes were selected using the FINDRISC-questionnaire. Parents’ and children’s energy balance-related behaviors data were collected by questionnaires at three time points (baseline, mid- and post intervention). Families assigned to the intervention group were invited to participate in a 2-year school-, community-, and family-based intervention to promote a healthier lifestyle, including counseling sessions (first intervention year) and text messages (second intervention year). Families assigned to the control group received standard care, including medical check-up results and recommendations and tips regarding a healthy lifestyle. To assess the intervention-effects, Mixed Models were conducted using the R-Package “lmer “with R v3.2.

**Results:**

Significant intervention effects were found on a certain number of families’ lifestyle behaviors. Significant favorable intervention effects were detected on parents’ water consumption and consumption of fruit and vegetables, and on children’s consumption of sweets and moderate-to-vigorous physical activity. Analyses by country revealed significant favorable intervention effects on water consumption and on moderate-to-vigorous physical activity in Belgian parents and on fruit and vegetable consumption among Belgian children, on sweets consumption among Spanish parents and children, and on moderate-to-vigorous physical activity among Finnish children. Unfavorable intervention effects were found on the consumption of soft drinks and sugar-containing juices among Hungarian children and parents, while when examining the intervention effects for the overall population and per country, 10 from the 112 investigated outcome variables were improved in the intervention group compared to the control group (9%).

**Conclusions:**

The Feel4Diabetes-intervention managed to improve a certain number of targeted lifestyle behaviors while the intervention was not effective on a large number of targeted lifestyle behaviors**.** The findings of the current study are encouraging, but further research is needed on how we can further improve effectiveness of lifestyle interventions to prevent type 2 diabetes in families at risk.

**Trial registration:**

The Feel4Diabetes-study is registered with the clinical trials registry http://clinicaltrials.gov, ID: 643708.

**Supplementary Information:**

The online version contains supplementary material available at 10.1186/s12966-021-01153-4.

## Background

Diabetes is one of the largest global health emergencies of the twenty-first century [1], with type 2 diabetes as the most common type, accounting for 90% of all diabetes cases [2]. In Europe, the number of adults with diabetes is estimated to be 8.8% of the population aged 20–79 years; and without sufficient actions this number is expected to raise to 9.8% by 2030 and to 10.3% by 2045 [2]. Undiagnosed type 2 diabetes results in a higher risk of diabetes-related complications such as eye and kidney disease, nerve and vascular damage, but also an increased healthcare use and related costs [2]. Type 2 diabetes is most commonly seen among (older) adults, but increasingly also children and adolescents are being diagnosed with type 2 diabetes due to rising levels of obesity [3]. Efforts to counter the raising trend of type 2 diabetes are, therefore, high on the public health agenda [4].

Type 2 diabetes is largely preventable through lifestyle changes. More specifically, improving lifestyle behaviors such as more physical activity (PA), less sedentary time and healthier eating behaviors can reduce the risk for developing type 2 diabetes [5–7]. Nevertheless, a high proportion of children and adults do not meet the age-specific recommendations for healthy lifestyle behaviors [8–14]. Therefore, there is urgency for effective lifestyle interventions to prevent type 2 diabetes in both children and adults. It is important to involve the entire family in lifestyle interventions, because family members tend to share a common environment and common attitudes towards health issues [15]. Additionally, identifying and targeting families at increased risk for developing type 2 diabetes further increases the (cost-)effectiveness of lifestyle interventions [2].

According to the socio-ecological model of health behavior, lifestyle behaviors are affected by factors at the personal, social, and environmental level [16]. Therefore, multilevel interventions, which include behavior change strategies in two or more levels, are recommended to enhance families’ healthy behaviors in order to prevent the development of type 2 diabetes [17–19]. Furthermore, literature showed that intervention strategies such as individual or group lifestyle counseling sessions and text messaging are effective strategies to reduce the risk of developing type 2 diabetes [20–25]. Multilevel interventions using counselling sessions to alter families’ lifestyle behaviors are currently underrepresented in literature.

Consequently, a two-year multilevel (school-, community- and family based) intervention, named Feel4Diabetes, was developed with the aim to prevent type 2 diabetes by promoting a healthy lifestyle in European families with an increased risk for developing type 2 diabetes. As indicated at the clinical trial registry, the main outcome of the Feel4Diabetes-study after one year was adults’ BMI. The Feel4Diabetes-intervention was jointly developed for six European countries [26].

The 1-year effects of this Feel4Diabetes-intervention on secondary outcome variables have already been reported [27]. The latter results only focused on at risk parents’ lifestyle behaviors (physical activity, sedentary behaviors and eating behaviors) and did not include the 2-year outcomes. The current study aimed to present the 2-year intervention results on both parents’ and their children’s lifestyle behaviors. However the data used in the current analysis were obtained from a questionnaire used for all families and delivered to parents via schools. Effects on subjectively measured PA, SB and eating behaviors were evaluated across the six European countries. Furthermore, country-specific intervention effects were also studied.

## Methods

### Study protocol

The Feel4Diabetes-study followed a theoretical framework based on the PRECEDE-PROCEED model to develop, implement and evaluate the intervention of which details have been published elsewhere [26]. The Feel4Diabetes-intervention was evaluated making use of a cluster randomized controlled trial design including intervention and control municipalities. The intervention was conducted in six European countries, representing three socio-economic levels derived from the World Bank’s Gross National Income (GNI) index [28] and the Eurostat’s Government Budget Deficit data in 2014 [29]: (1) high-income countries (Belgium and Finland), (2) low-to-middle-income countries (Bulgaria and Hungary) and (3) high-income countries under austerity measures (Greece and Spain). As the prevalence of type 2 diabetes is significantly higher in (every area of) low- to-middle-income countries [30], every area in Bulgaria and Hungary was defined as a vulnerable area. In high-income countries and high-income countries under austerity measures, the prevalence of type 2 diabetes is higher in low SES areas [2], and therefore only low SES areas were defined as vulnerable areas in Belgium, Finland, Greece and Spain. To select the low SES areas, all municipalities in the selected provinces were divided into tertiles based on socioeconomic indices retrieved from official sources and authorities (i.e. literacy or unemployment rates) [31–34]. Municipalities within the tertile of the lowest SES indices were included in the study. In all countries, lists of all primary schools within the selected vulnerable areas were created and primary schools were randomly selected and recruited from each area until the recruitment goal was met. In total, the headmasters of 219 primary schools (response rate = 40.2%) confirmed their participation. All parents of primary schoolchildren from the first, second and third year (aged 6–9 years) received an informed consent, the FINDRISC-Questionnaire (Finnish Diabetes Risk Score-Questionnaire; a tool that assesses the 10-year risk of developing type 2 diabetes) [35, 36] and the Energy Balance Related Behaviors Questionnaire (EBRB-Questionnaire) developed for the Feel4Diabetes study. In total, 11,396 families confirmed the participation of their family (child and at least one parent) by signing the informed consent and filling out both questionnaires. After the completion of baseline measurements, municipalities were assigned to the intervention or standard care group (ratio 1:1).

For the present study, only families with an increased risk for developing type 2 diabetes were selected (parents and their children; *n* = 4484 families (39.3%)), based on the parent’s score on the FINDRISC-questionnaire. A family was considered being at high risk if at least one of the parents got a score of 9 points or more (selection criteria: see methods-measurements). In total 2537 families confirmed their participation in the high-risk measurements. Children’s and high-risk parents’ height and weight were objectively measured in school (children) and local municipality centers or home setting (parents). Measurements were conducted by trained researchers, using standardized protocols and calibrated equipment [37] between April and June 2016 (baseline), April and June 2017 (Mid-intervention) and April and June 2018 (Follow up).

### The Feel4Diabetes-intervention

The multilevel Feel4Diabetes-intervention was implemented for two school years (September 2016–April 2017 and September 2017–April 2018) and involved three different levels, namely the school-, the community- and the family level which are described in detail below. All levels focused on changes in the school, home and local community environment in order to adopt a healthier and more active lifestyle among families. The general goals of the Feel4Diabetes-intervention were: to increase the consumption of water (instead of sugar-sweetened drinks), fruits and vegetables (instead of unhealthy snacks) and breakfast, to increase physical activity and to decrease sedentary time. The Feel4Diabetes-intervention was developed jointly for the six countries, but each country was advised to make adaptations to local needs and contextual circumstances.

#### Family level

During the first intervention year, high-risk parents from the intervention group were invited to participate in six counseling sessions aiming to inform families on risk factors related to type 2 diabetes and to encourage them to adopt a healthier lifestyle. The counselling sessions included behavioral change techniques aiming to increase motivation and self-efficacy, improve their self-regulation and set goals to adopt a healthier and more active lifestyle. A more detailed description of the content of the counseling sessions can be found elsewhere [26, 27].

The intervention during the second year (2017–2018) aimed to maintain the changes achieved during the first intervention year and therefore, it was less intensive compared to the first year. During this second year, parents received motivational guidance via text messages sent to their mobile phones. Text messages were created by researchers and were related to the following themes: (1) moderately active everyday life (e.g. “You can play hide-and-seek or make a fitness park in your nearby woods. Use your imagination together!”), (2) physical activity increase (e.g. “Do something physically active with your family today! Go for a walk or play together”), (3) reduce sedentary time (e.g. “An easy habit to sit less: stand while talking on the phone”) (4) good carbs (e.g. “A rainy day? Making oatmeal cookies together, fun and healthy”), (5) Healthy fats (e.g. “If you drink 2 glasses of milk daily and switch from regular to skimmed milk, you cut down your fat intake by over 2kg per year. Skimmed milk is also good for children) (6) fruit and vegetables (e.g. “We tend to eat more foods that are visible and on reach. Therefore, it is wise to keep only healthy foods, like fruit, on sight.”) and (7) regular meal pattern (e.g. “Eat like a king in the morning, like a prince in the afternoon, and like a beggar in the evening. When you consume most of energy by afternoon, you increase your vigor.”). Each country could adapt the text messages to country specific needs and contextual circumstances. Between September and November 2017, parents were invited to the second intervention year during a seventh counseling session. After parents’ confirmation to participate in the second intervention year, they received text messages with questions regarding satisfaction with their current lifestyle and regarding self-efficacy to change lifestyle behaviors. Thereafter, parents selected one of the health-related themes and received two motivational text messages per week related to their chosen theme. Every eight weeks, parents were given the opportunity to choose another theme.

Families in the control municipalities received their medical check-up results (parents’ blood indices, parents’ and children’s Body Mass Index (BMI) and step counts) and were offered one counseling session on lifestyle changes, delivered by trained researchers. Furthermore, they received an extensive leaflet with easy to read recommendations and tips to adopt a healthy lifestyle among their family (further referred to as standard care).

#### School level

At the beginning of both intervention years (September 2016 and September 2017), primary school teachers and head masters in the intervention schools received an information session on how they could create a more supportive social and physical environment that promotes a healthy and active lifestyle for children at the school setting. In these sessions, feasible opportunities to promote healthy behaviors among children and how teachers could act as a role model in the school setting were illustrated by researchers. Additionally, in the first intervention period, newsletters for parents were distributed to all participating children. These newsletters aimed to inform and actively engage families in the intervention group. Control schools were asked to continue with the standard curriculum [26].

#### Community level

In the first and second intervention year, available infrastructure and existing health-related activities in the neighborhood for both children and parents were communicated via newsletters or other means of communication (such as a private Facebook group) aiming to promote a healthy and active lifestyle in the participating families. These opportunities were identified by local researchers in collaboration with local municipality authorities. Examples of available infrastructure were access to sports halls and parks or school yards after school hours. Examples of health-related activities were local walks, information sessions “Health- and Fitness apps”, etc. Families of the standard care group did not receive this community-based intervention.

### Measurements

#### Diabetes risk score

The FINDRISC-Questionnaire is a validated screening tool for predicting the risk of type 2 diabetes, including eight questions on age, BMI, waist circumference, PA, daily consumption of fruit, berries or vegetables, history of antihypertensive drug treatment, history of high blood glucose and family history of diabetes [38]. Because a FINDRISC-score of at least 9 points identifies more than 70% of incident cases of type 2 diabetes, this score is often used to identify individuals at risk for developing type 2 diabetes [38]. A family was considered being at high risk if at least one of the parents got 9 points or more.

#### Subjectively measured lifestyle behaviors

Parents filled in questionnaires about their and their children’s energy balance related behaviors. Moderate-to-vigorous PA (MVPA) was subjectively measured by the following questions: “How many days during the last week did you (parent) spend in MVPA for a total of at least 30 minutes per day?” and “How many days during the last week did your child spend in MVPA for a total of at least 1 hour per day?”. Parents’ and children’s screen-time behavior during the week was assessed by the following question: “About how many hours per day do you (parent) and your child usually devote to screen-activities (excluding school/work)?” Answer options (categorical values) were expressed in hours per day. Answers options: None, < 30 min/day, 30 min to < 1 h/day, 1 to < 2 h/day, 2 to < 3 h/day, …, ≥ 7 h/day. Afterwards, these categorical values were recoded into numerical values, expressed in minutes per day. Consumption of water, soft drinks and juices containing sugar, fruit and vegetables, unhealthy snacks and breakfast were assessed. The general question was: “Indicate how often you (parent) and your child consume: water, soft drinks and juices with added sugar, fruit/berries (fresh or frozen), fruit and berries (canned or dried), vegetables, sweets, salty snacks/fast-food”. Answer options (categorical values) were expressed in portions per week. Answer options: less than 1 time/week, 1 or 2 times/week, 3 or 4 times/week, 5 or 6 times/week, 1 or 2 times/day, 3 or 4 times/day, 5 or 6 times/day, > 6 times/day. These categorical values were recoded into numerical values expressed in portions per day. To assess the total consumption of fruit and vegetables per day, the daily consumption of fruit and berries (fresh or frozen), fruit and berries (canned or dried) and vegetables were summed. Outliers for the total consumption of fruit and vegetables per day (defined as values above three standard deviations from the mean) were capped and reassigned the value of the mean plus three standard deviations. The daily breakfast consumption was measured by the following questions. “How many days do you/does your child usually eat breakfast?” separately for weekdays and weekend days. The number of days consuming breakfast on weekdays and weekend days were summed. The reliability of the questionnaires regarding these lifestyle behaviors was evaluated and was found to be acceptable [39].

#### Socio-demographic variables

Parents reported their date of birth, sex and educational level (years of education), as well as their child’s day of birth and sex. Parental education level was recoded into a low parental education (defined as ≤14 years of education) and a high parental education (defined as > 14 years of education) [40]. As in European educational systems, more than 14 years of education implies attendance of higher education (e.g., bachelor program). Family education was categorized as follows: low (both parents having low education), medium (one of the parents having low education), high (both parents having a high education) [40].

### Statistical analyses

All data obtained during the Feel4Diabetes measurement periods were used in the analyses. Descriptive statistics for the sample demographics and the attendance rates during the counseling sessions were computed using SPSS statistics 25.0 for Windows (SPSS Inc., Chicago, IL). To investigate the differences in sociodemographic factors between the intervention and standard care group, Mann-Whitney U tests and Chi^2^ tests were conducted in SPSS. To assess the effectiveness of the Feel4Diabetes-intervention, pre-defined intervention effects were analyzed across all countries, and per country. The intervention effects were investigated over 2 intervention years. Beta and standard errors were reported from baseline to mid-intervention (after one year) and from baseline to post-intervention (after two years). The intervention effects were tested by Linear Mixed Models with a random intercept (measurements nested within a participant) and three terms: group (intervention versus control group), time (baseline, mid-intervention and post-intervention) and the interaction term between those two variables. No covariates were included in the analyses. Statistically significant intervention effects (interaction term with *p*-value < 0.05) were reported and illustrated with model effect plots, which included means and 95% confidence intervals of the means [41]. The models were conducted and plotted with the R-packages “lme4 “and “effects”, respectively; using R version 3.2 (R Core Team 2016, R Foundation for Statistical Computing, Vienna, Austria, http://www.r-project.org).

## Results

### Sample characteristics

In total 2418 parents from high-risk families (1329 parents from intervention group and 1089 parents from standard care group) filled out the EBRB-questionnaire for themselves and their child included in the study.

**Fig. 1:**
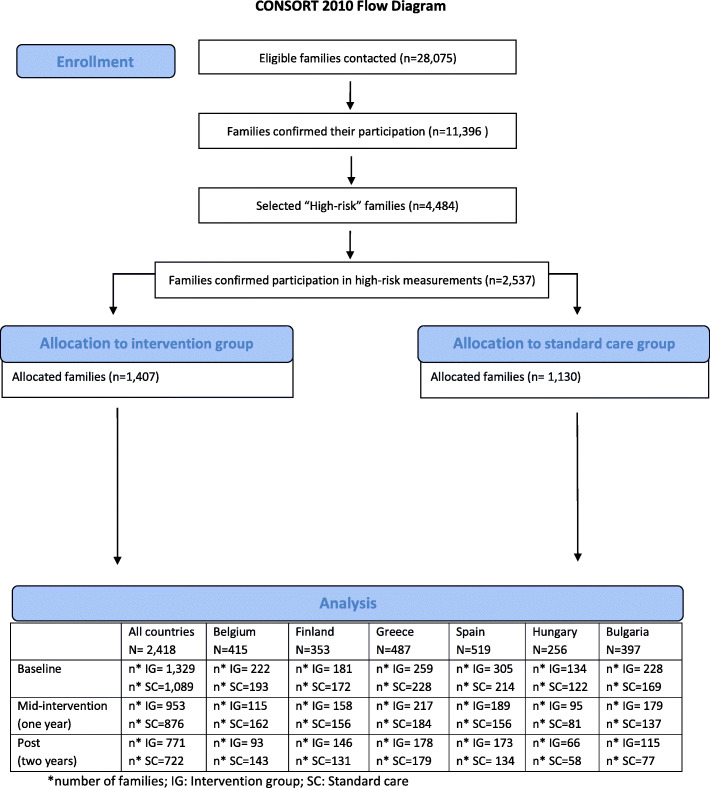
CONSORT flow diagram

Parents were on average 40.1 years old (standard deviation (SD) = 5.50) and had a mean BMI of 26.7 kg/m^2^ (SD = 5.10) at baseline. In total, 92.0% of them were female caregivers and 37.8% were lower educated. At baseline, the children were on average 8.1 years (SD = 1.0), had a mean BMI z-score of 0.68 (SD = 1.11) and 51.0% of the included children were girls. Parents’ FINDRISC-score ranged between 0 and 22 points, with a mean of 9.7 points (SD = 4.50). Family’s FINDRISC-score, i.e. the highest score among the parents, ranged between 9 and 24 with a mean of 12.4 points (SD = 2.90). At baseline, no significant differences were found the sociodemographic factors (age, sex, BMI (z-score), (family)education and (parents’) FINDRISC-score between the intervention and standard care group. The CONSORT flow diagram for participants throughout the study can be found in Fig. 1. All intervention effects (significant and non-significant intervention effects) can however be found in supplementary file 1. Below, only the significant intervention effects were reported.

### Attendance rates during the Feel4Diabetes-intervention

At the family level, the attendance rates during each counseling session were recorded and can be found within Table 1. At school level, all participating children received the intervention and all participating parents received the newsletters distributed via schools.

**Table 1:** Attendance rates during the first and second intervention year

Number of counseling session	all countries	Belgium	Finland	Greece	Spain	Hungary	Bulgaria
1	**61.6%**	88.7%	67.3%	74.4%	46.5%	28.5%*	98.7%
2	**48.6%**	23.6%	57.9%	72.5%	36.6%	34.3%*	89.7%
3	**42.6%**	40.4%	64.5%	60.6%	25.4%	23.7%*	49.4%
4	**38.2%**	25.8%	39.7%	60.1%	25.2%	28.5%*	34.2%
5	**36.5%**	22.2%	58.9%	56.3%	19.3%	27.0%*	35.4%
6	**33.3%**	15.3%	54.2%	54.3%	15.7%	24.1%*	26.6%
7	**40.0%**	5.5%	54.7%	48.8%	36.4%	35.9%*	51.9%

*60.4% of the data is missing

### Effectiveness of the Feel4Diabetes-intervention on subjectively measured lifestyle behaviors

When examining the intervention effects for the overall population and per country, 10 from the 112 investigated outcome variables were improved in the intervention group compared to the control group (9%).

#### Water consumption

**Fig. 2:**
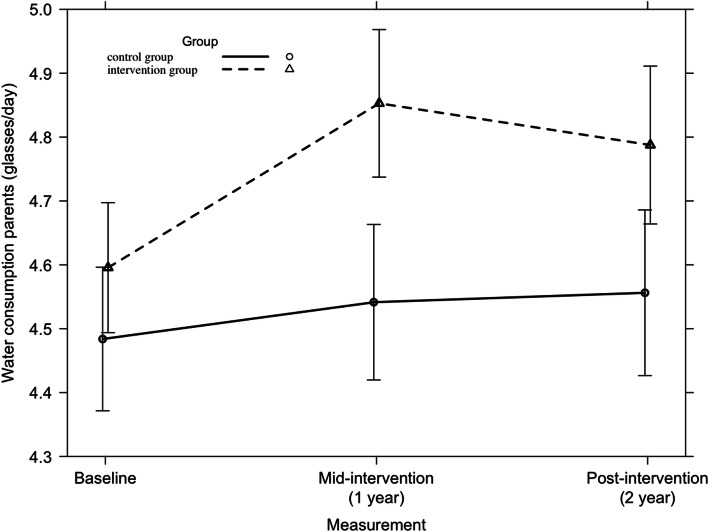
Parents’ water consumption from baseline to mid-intervention to post-intervention across all countries

**Fig. 3:**
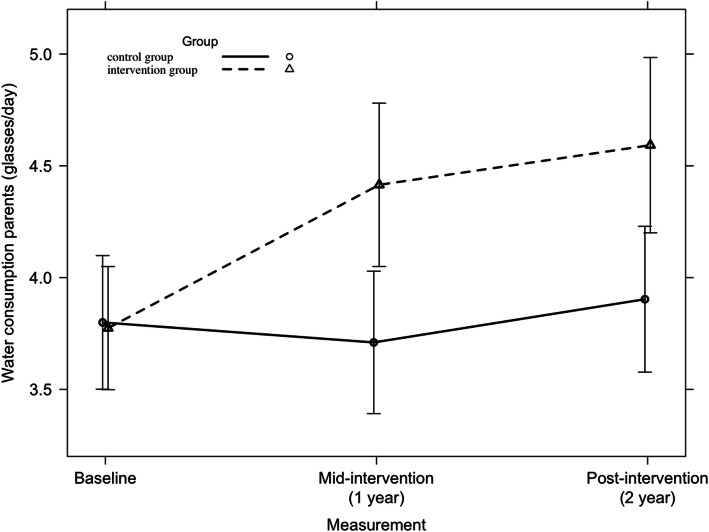
Water consumption among Belgian adults from baseline to mid-intervention to post-intervention across all countries

Across all countries, a significant 2-year intervention effect was detected on water consumption among parents (F = 3.24, *p* = 0.04), which is represented in Fig. 2. When looking at country level, a significant 2-year intervention effect was found (F = 6.28, *p* = 0.002) among Belgian adults (Fig. 3). Among Greek parents only in the first intervention year a larger increase between baseline and mid-intervention was found in the intervention group compared to the standard care group (β(SE) = + 0.3 glasses/day (0.14)). No significant 2-year intervention effect was detected.

Among children, no significant 2-year intervention effect on water consumption could be detected across the participating countries and no significant country-specific intervention effects were found (all *p* > 0.05).

#### Fruit and vegetable consumption

**Fig. 4:**
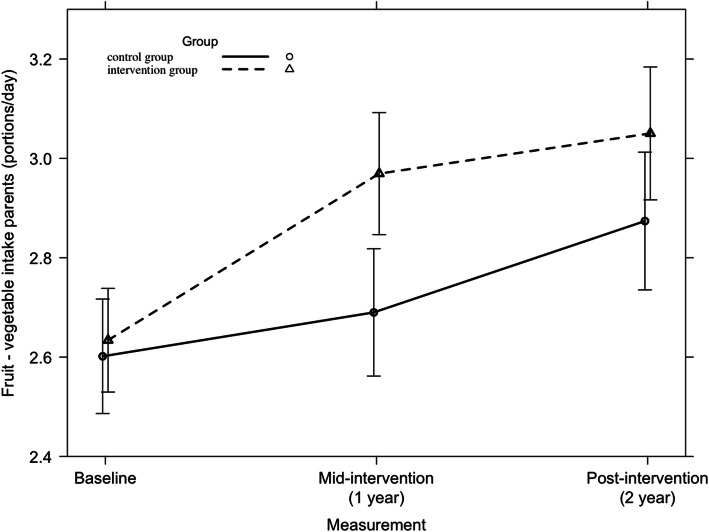
Parents’ fruit and vegetable consumption from baseline to mid-intervention to post-intervention across all countries

A significant 2-year intervention effect was found on the consumption of fruit and vegetables among parents across all countries (F = 3.31, *p* = 0.04), which is presented in Fig. 4. No country-specific significant 2-year intervention effects could be detected on parents’ fruit and vegetable consumption.

**Fig. 5:**
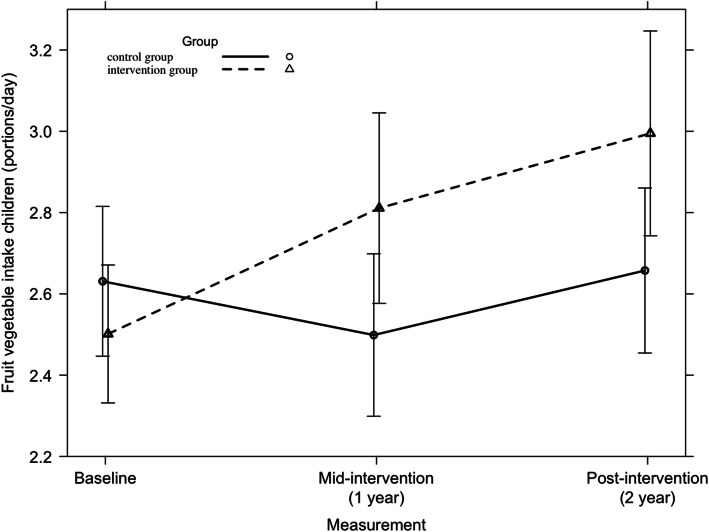
Fruit and vegetable consumption among Belgian children from baseline to mid-intervention to post-intervention

Across all countries, no significant 2-year intervention effect was found on children’s consumption of fruit and vegetables (F = 1.44, *p* = 0.24). Among Belgian children, a significant 2-year intervention effect was found (F = 5.43, *p* = 0.01), which is presented in Fig. 5. Within the other countries, no significant 2-year intervention effects on children’s fruit and vegetable consumption were detected.

#### Consumption of sweets

**Fig. 6:**
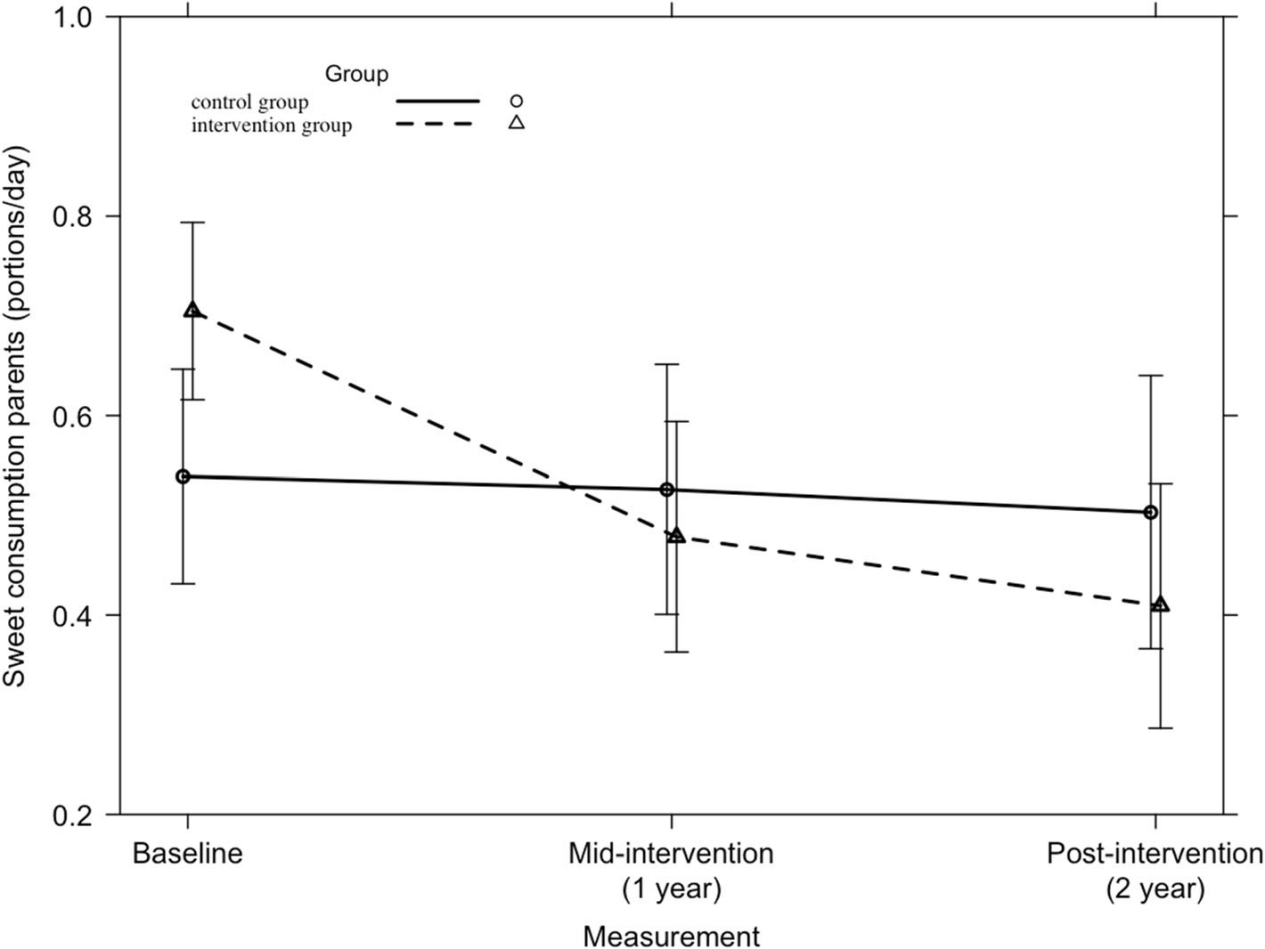
Consumption of sweets among Spanish parents from baseline to mid-intervention to post-intervention

Overall, no significant 2-year intervention-effect was found on the consumption of sweets among parents (F = 1.51, *p* = 0.22). Among Spanish parents, a significant 2-year intervention effect was found on the consumption of sweets (F = 3.66; *p* = 0.03). This is presented in Fig. 6. Among Finnish parents, no 2-year intervention effect was found (*p* = 0.04). Only in the first year, the intervention group had a higher decrease in consumption of sweets compared to the standard care group (β (SE) = − 0.09 portions per day (0.05)). For the other participating countries, no significant 2-year intervention effects on parents’ consumption of sweets were found.

**Fig. 7:**
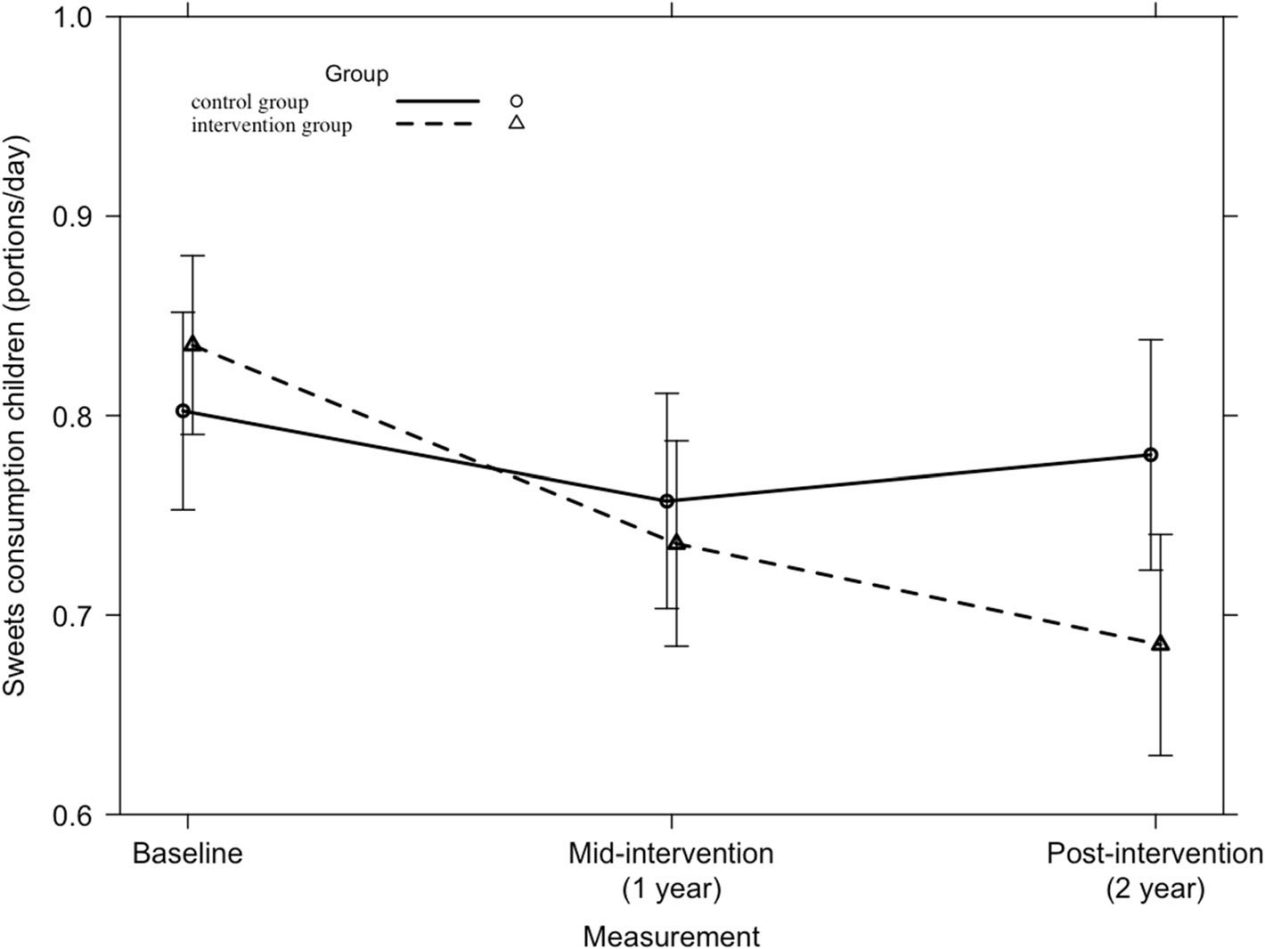
Children’s consumption of sweets from baseline to mid-intervention to post-intervention across all countries

**Fig. 8:**
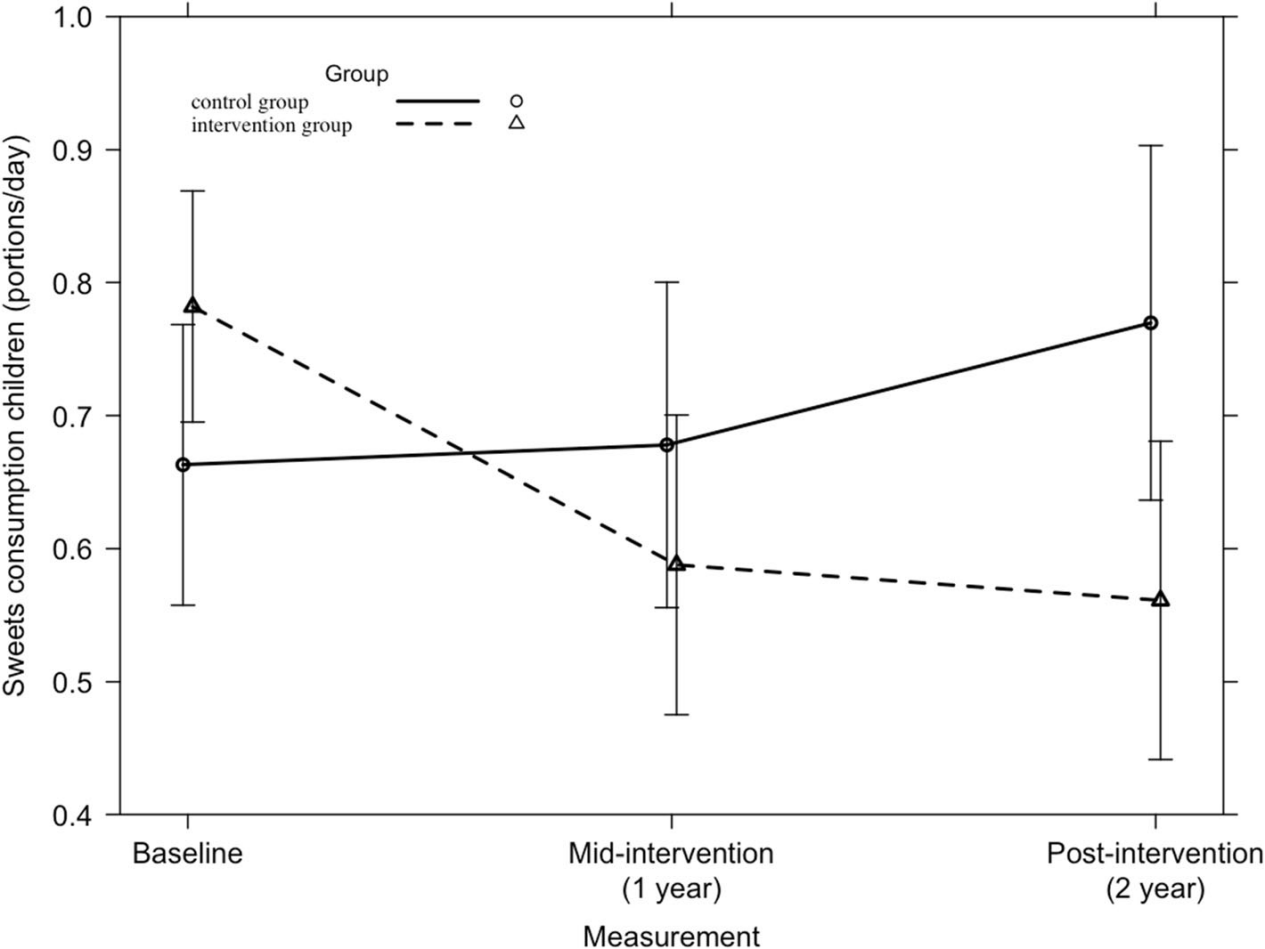
Consumption of sweets among Spanish children from baseline to mid-intervention to post-intervention

Across all participating countries, a significant 2-year intervention effect was found on the consumption of sweets among children (F = 5.13, *p* = 0.01). This is represented in Fig. 7. A significant country-specific 2-year intervention effect was found on the consumption of sweets of Spanish children (F = 5.45, *p* = 0.005) (Fig. 8). No other country-specific 2-year intervention effects on children’s consumption of sweets were found.

#### Consumption of soft drinks and juices containing sugar

**Fig. 9:**
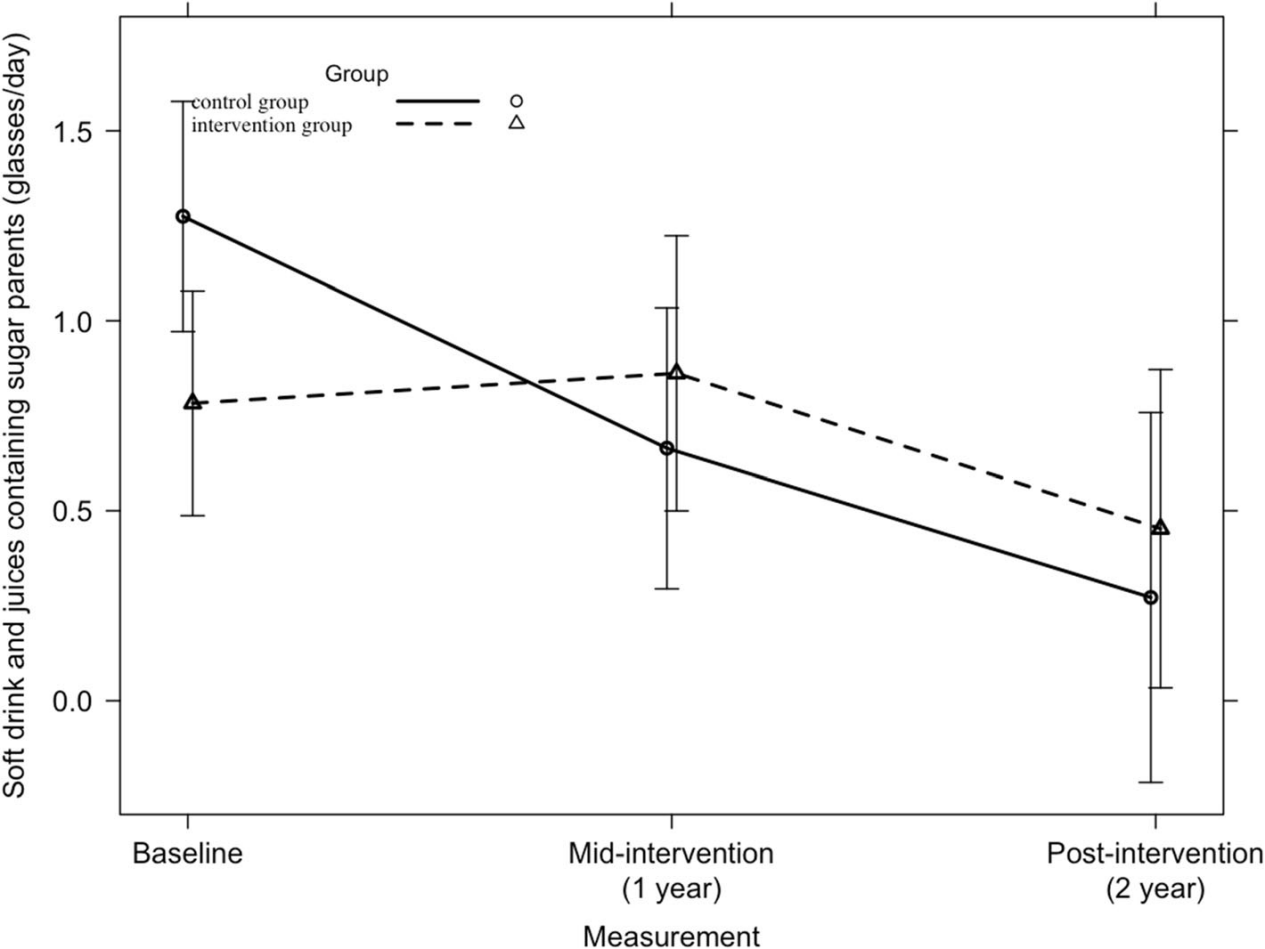
Consumption of soft drinks and juices containing sugar among Hungarian parents from baseline to mid-intervention to post-intervention across all countries

Across all countries, no significant 2-year intervention effect was found on parents’ consumption of soft drinks and sugar-containing juices. Among Hungarian parents, a small but significant unfavorable 2-year intervention effect was found (F = 3.48, *p* = 0.03), which is represented in Fig. 9. Furthermore, no other significant country-specific 2-year intervention effects were found on the consumption of soft drinks and juices containing sugar among parents.

**Fig. 10:**
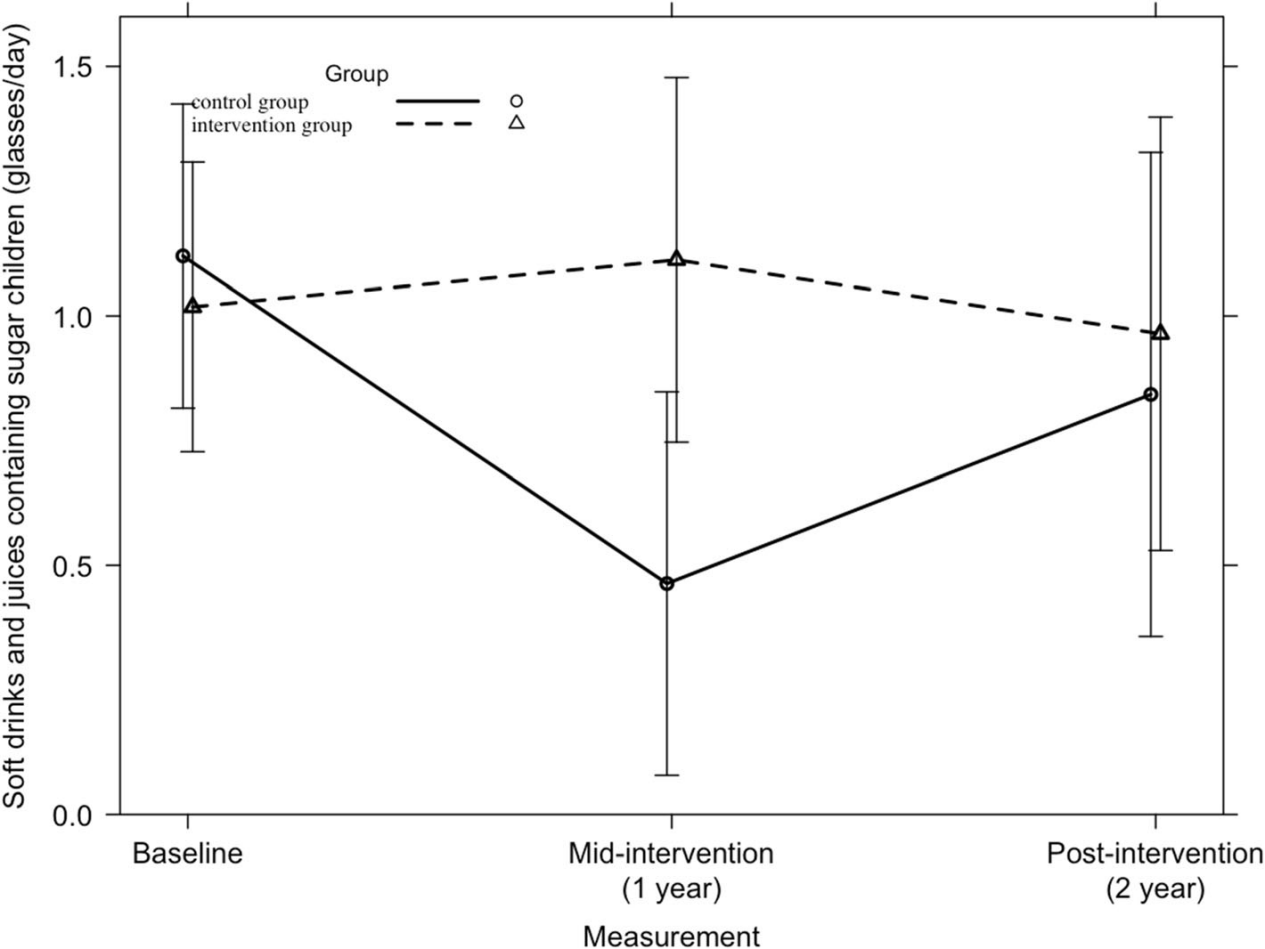
Consumption of soft drinks and juices containing sugar among Hungarian children from baseline to mid-intervention to post-intervention

Across all countries, no significant 2-year intervention effect was found on children’s consumption of soft drinks and juices containing sugar (F = 0.33, *p* = 0.72). Among Hungarian children, a small significant unfavorable intervention effect was found, which is represented in Fig. 10 (F = 3.16, *p* = 0.04). Among Spanish children, a significant first year intervention-effect was found on the consumption of soft drinks and juices containing sugar (p = 0.04). However, no significant 2-year intervention effect could be detected (F = 2.24, *p* = 0.11). Further, no significant intervention effects were found on children’s soft drinks and juices containing sugar in the other countries.

#### Salty snacks and fast food

Across all countries, no significant 2-year intervention effect was found on the consumption of salty snacks and fast food among parents (F = 0.40, *p* = 0.67) or children (F = 2.89, *p* = 0.06) and also no country-specific 2-year intervention effects were detected.

#### Breakfast consumption

Overall, no significant 2-year intervention effect was found on parents’ breakfast consumption (F = 1.24, *p* = 0.29) and no significant 2-year intervention effects were found, separately for each country. Further, no significant 2-year intervention effects were detected on children’s breakfast consumption across the countries (F = 0.75, *p* = 0.47) and also no significant 2-year intervention effects were found separately for each country.

#### Moderate-to-vigorous physical activity

**Fig. 11:**
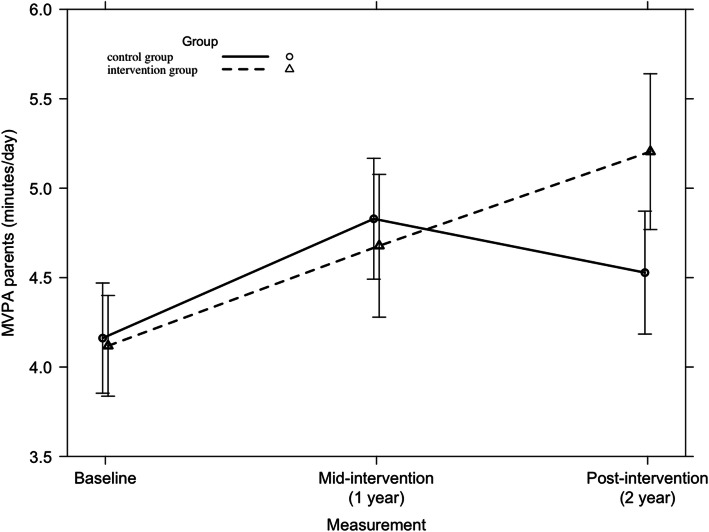
Moderate-to-vigorous physical activity among Belgian parents from baseline to mid-intervention to post-intervention

Across the countries, no significant 2-year intervention effect was found on parents’ MVPA (F = 2.42, *p* = 0.09). A significant 2-year intervention effect was found in Belgian parents (F = 3.87, *p* = 0.022). This is represented in Fig. 11. No other significant country-specific 2-year intervention effects were found on parents’ MVPA.

**Fig. 12:**
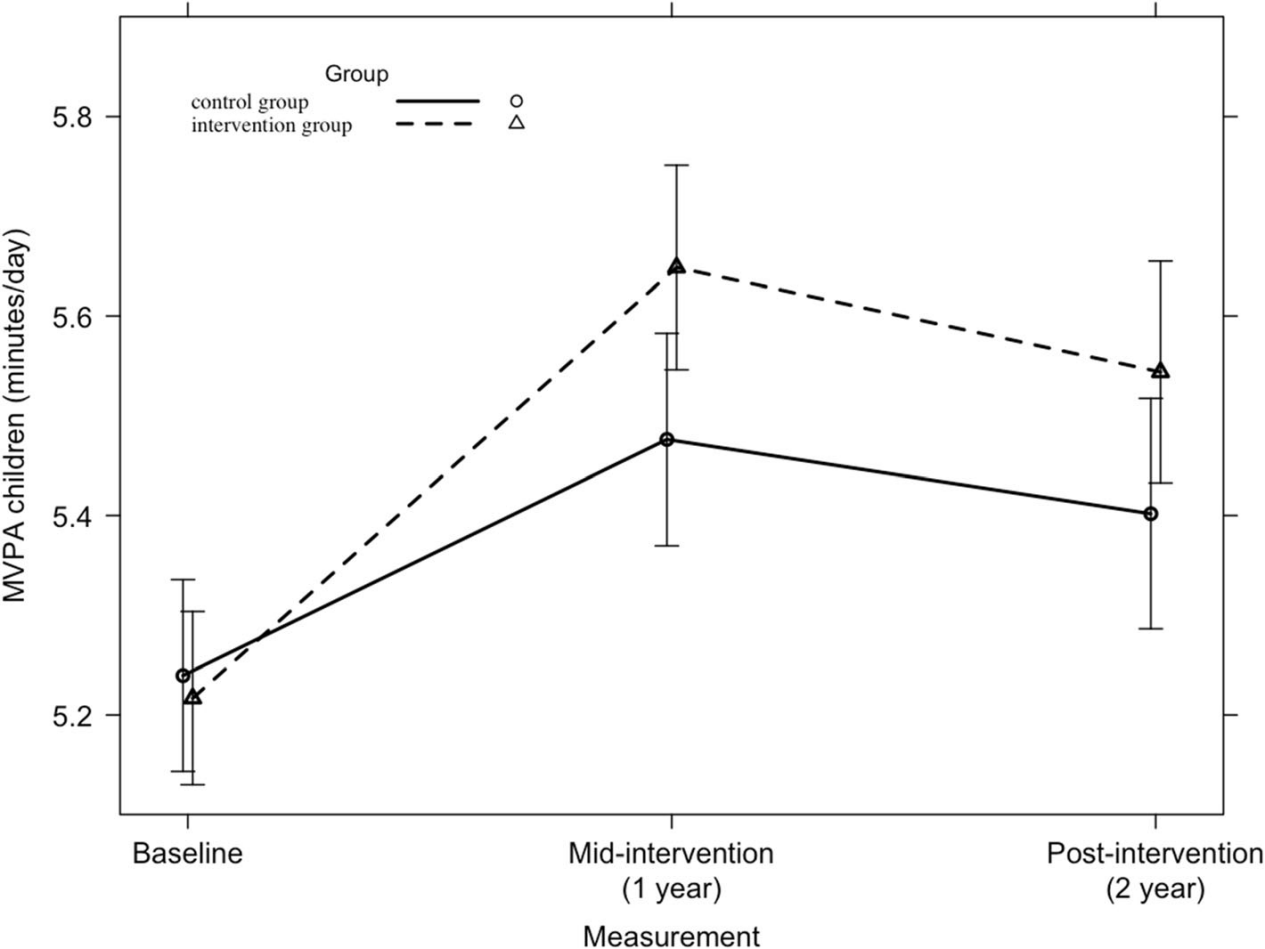
Moderate-to-vigorous physical activity among children from baseline to mid-intervention to post-intervention across all countries

**Fig. 13:**
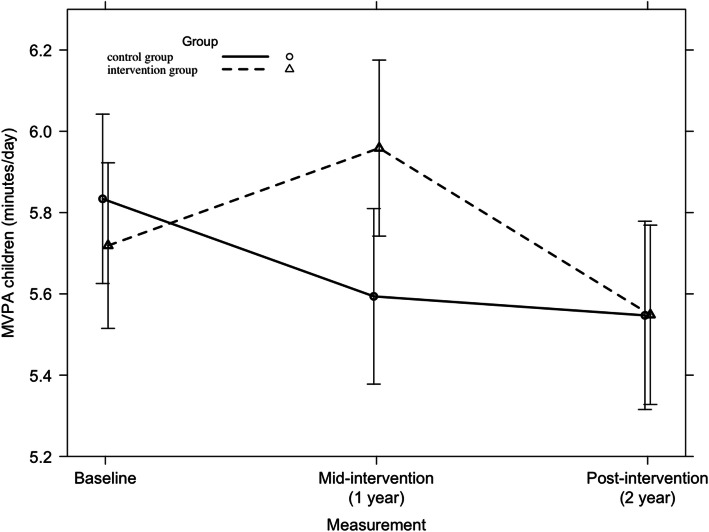
Moderate-to-vigorous physical activity among Finnish children from baseline to mid-intervention to post-intervention

Among children, a significant 2-year intervention effect was found on MVPA across all countries (F = 3.37, *p* = 0.03) and in Finland (F = 4.52, *p* = 0.010), which is represented in Figs. 12 and 13 respectively. Further, no significant country-specific intervention effects were found on children’s MVPA.

#### Screen-time

No significant 2-year intervention effect was found in time spent in front of a screen across the countries among parents (F = 1.28, *p* = 0.30) or children (F = 0.95, *p* = 0.39) and also no country-specific intervention effects were found.

An overview of the significant intervention effects can be found in Table 2 and in Additional Table 1 all results of the longitudinal analyses are presented.

**Table 2:** Overview of significant intervention effects on self-reported lifestyle behaviors

		Total number of observations (n)	Group	Baseline Mean (SD)	Mid-intervention Mean (SD)	Post-intervention Mean (SD)	Time x condition (F-value)	Baseline – Mid-intervention β(SE)	Baseline – Post-intervention β(SE)
**Parents**									
All countries	Water (glasses/day)	5299	I	4.6 (1.90)	4.9 (1.71)	4.8 (1.72)	3.24*	0.20 (0.08) *	0.12 (0.09)
			SC	4.5 (1.94)	4.5 (1.90)	4.5 (1.88)			
	F&V (portions/day)	5341	I	2.7 (1.86)	3.0 (1.94)	3.1 (2.03)	3.31*	0.25 (0.10) *	0.14 (0.10)
			SC	2.6 (1.84)	2.7 (1.92)	2.9 (1.90)			
Belgium	Water (glasses/day)	846	I	3.8 (2.17)	4.5 (1.83)	4.9 (1.77)	6.28**	0.73 (0.24) **	0.72 (0.25) **
			SC	3.8 (2.13)	3.8 (2.01)	4.0 (1.88)			
	MVPA (days of 30 mins MVPA/day)	869	I	3.9 (2.34)	4.2 (2.14)	4.3 (2.16)	3.87*	−0.11 (0.28)	0.72 (0.30) *
			SC	3.9 (2.28)	4.1 (2.22)	4.0 (2.22)			
Finland	Sweets (portions/ /day)	837	I	0.5 (0.49)	0.4 (0.31)	0.4 (0.38)	2.09	−0.09 (0.05) *	−0.03 (0.05)
			SC	0.5 (0.38)	0.5 (0.38)	0.5 (0.40)			
Greece	Water (glasses/day)	1156	I	4.6 (1.82)	4.9 (1.71)	4.8 (1.76)	0.65	0.30 (0.14) *	0.26 (0.15)
			SC	4.6 (1.91)	4.6 (1.84)	4.6 (1.93)			
Spain	Sweets (portions/day)	1033	I	0.7 (0.90)	0.5 (0.71)	0.4 (0.61)	3.66*	−0.21 (0.10) *	−0.26 (0.12) *
			SC	0.5 (0.52)	0.5 (0.95)	0.5 (0.69)			
Hungary	Soft drink and juices containing sugar (glasses/day)	452	I	0.8 (1.48)	0.9 (1.75)	0.4 (0.80)	3.48*	0.69 (0.29) *	0.67 (0.35)
			SC	1.3 (2.17)	0.5 (1.37)	0.3 (0.37)			
**Children**									
All countries	Consumption of sweets	5306	I	0.8 (0.89)	0.7 (0.77)	0.6 (0.66)	5.13**	−0.05 (0.04)	−0.13 (0.04)**
			SC	0.8 (0.84)	0.7 (0.75)	0.8 (0.77)			
	MVPA (days of 60 min MPA/day)	5357	I	5.2 (1.68)	5.7 (1.44)	5.6 (1.49)	3.37*	0.20 (0.08) *	0.17 (0.09)
			SC	5.2 (1.68)	5.5 (1.60)	5.4 (1.53)			
Belgium	F&V (portions/day)	868	I	2.5 (1.32)	2.8 (1.35)	3.0 (1.32)	5.43**	0.44 (0.16) **	0.47 (0.17) **
			SC	2.6 (1.26)	2.5 (1.06)	2.7 (1.31)			
Finland	MVPA (in days of 60 mins MVPA/day)	891	I	5.7 (1.36)	6.0 (1.24)	5.6 (1.49)	4.52*	0.48 (0.17) **	0.12 (0.17)
			SC	5.8 (1.31)	5.6 (1.42)	5.5 (1.44)			
Spain	Sweets (portions/day)	1038	I	0.8 (0.86)	0.6 (0.59)	0.5 (0.66)	5.45**	−0.21 (0.10) *	−0.33 (0.10) **
			SC	0.7 (0.56)	0.7 (0.85)	0.8 (0.84)			
	Soft drinks and juices containing sugar (glasses/day)	1019	I	0.4 (0.75)	0.1 (0.35)	0.2 (0.70)	2.24	0.10 (−0.17) *	−0.07 (0.09)
			SC	0.3 (0.47)	0.2 (0.63)	0.2 (0.56)			
Hungary	Soft drinks and juices containing sugar (glasses/day)	454	I	1.1 (1.75)	1.1 (1.82)	0.8 (1.07)	3.16*	0.75 (0.30) *	0.22 (0.36)
			SC	1.1 (1.98)	0.4 (0.84)	0.8 (1.06)			

*p-value < 0.05; **p-value< 0.01; I Intervention group; C Control group; SD Standard deviation; β(SE): Beta (Standard error); MVPA moderate-to-vigorous physical activity. One portion size: water and soft drinks/juices containing sugar: 2.5 dl, fruit and vegetables: 1/2 cup (2.5 dl) or the size of a tennis ball, sweets: a chocolate bar, half a cup of sweets, cookies or one scoop of ice-cream, salty/snacks fast food: a small hamburger, a small bag of chips or a slice of pizza.; I: intervention group; SC standard care; F&V: fruit & vegetable

## Discussion

Results of the current study revealed that significant intervention effects could be detected for a certain number of lifestyle behaviors across and also within the six countries. Overall, the 2-year Feel4Diabetes-intervention was effective in improving the water consumption and the consumption of fruit and vegetables among parents. Among children, the intervention was effective in reducing the consumption of sweets and improving PA. Also some country-specific intervention effects were found. In Belgium, significant intervention effects were found on parents’ water consumption, on parents’ MVPA and on children’s fruit and vegetable consumption. In Spain, significant intervention effects were found on parents’ and children’s consumption of sweets; and in Finland a significant intervention effect was found in children’s MVPA. Unfortunately, in Hungary unfavorable intervention effects were found on parents’ and children’s consumption of soft drinks and juices containing sugar. During the first intervention year, the Feel4Diabetes-intervention aimed to improve type 2 diabetes related lifestyle behaviors by intervening at family, school and community level, while during the second year, the intervention aimed to maintain the changes achieved during the first intervention year. This intervention-approach was clearly reflected in most significant intervention effects of the current study: the intervention group mainly improved from baseline to mid-intervention and afterwards, a stagnation or limited decrease was found from mid-intervention to post-intervention. Based on the above-mentioned significant intervention effects, it can be concluded that providing the Feel4Diabetes lifestyle intervention was more effective compared to standard care for certain lifestyle behaviors.

Although the Feel4Diabetes-intervention improved some lifestyle behaviors, the large majority of lifestyle behaviors (91%) could not be enhanced**.** More specifically, the consumption of sugar-sweetened beverages, salty snacks and fastfood, daily breakfast consumption and screen-time could not be improved in the intervention group compared to the control group. In addition, no significant favorable intervention effects were found in Bulgaria, Hungary and Greece. Several factors of the Feel4Diabetes-study could underly these non-significant intervention effects. First, a critical reflection on the content of the Feel4Diabetes-intervention was made. In general, to save costs and enhance feasibility, the Feel4Diabetes counseling sessions were less intensive (7 counseling sessions in 12 months), compared to some effective lifestyle interventions conducted in previous research [22, 23, 42–46]. Therefore, more research is needed to find the optimal balance in order to develop cost-effective lifestyle interventions for families at risk for type 2 diabetes. Furthermore, during the second Feel4Diabetes-intervention year, mobile text messages were provided aiming to keep participants motivated to maintain positive lifestyle changes made during the first intervention year. Previous research already showed that mobile phone messaging is an inexpensive [25], but effective tool to intervene on health in at risk individuals [24, 47]. Also, within the Feel4Diabetes-intervention, mobile text messaging seems to be a promising tool in maintaining the improvements in most lifestyle behaviors made during the first intervention year. However, the intervention effects on some lifestyle behaviors (water consumption among Greek parents, consumption of sweets in Finnish parents and the consumption of soft drinks and juices containing sugar in Spanish children) observed at the mid-intervention measurement did not seem to persist until the post-test measure. This finding might be due to the limited number of themes provided to participants in the text messages. During the second intervention year, families did not receive support and encouragement regarding the consumption of soft drinks and juices containing sugar and the consumption of sweets. Therefore, for future research, it might be better to provide a wider range of motivational text messages, responding to all targeted behaviors in the first intervention year.

The attendance rate is a second factor that might have caused the limited observed behavioral changes that was intended by the intervention. Throughout the Feel4Diabetes-intervention, low attendance rates were reported from baseline to post-intervention, which was also seen in other studies focusing on individuals at risk for developing type 2 diabetes [48, 49]. To maximize the attendance of the Feel4diabetes-intervention at family level, the most intensive part of the intervention, several efforts were made. As recommended in previous research [50–52], incentives were provided in some countries (e.g. breakfast was provided in Belgium), counseling sessions were organized at an accessible location, parents were reminded to attend the sessions by a mobile text message, and/or childcare was provided. Despite these efforts, the number of attended counseling sessions and the number of individuals participating in the second intervention year were low and clear differences could be detected across the countries. Up to now, the reasons for these high-drop out figures are unknown. A process evaluation should be conducted in order to find the reasons for the high drop out. At the community level, available infrastructure and existing low-cost activities in the neighborhood were promoted. However, the intensity of families’ participation was not recorded within the current study, so no conclusion can be drawn based on the attendance rate of participants at community level. Lack of time and lack of motivation are two main barriers for lifestyle change in families at risk for developing type 2 diabetes [53–55]. First, E-health interventions are recommended for future research due to their flexibility in time. E-health interventions have proven to be effective on weight loss in previous diabetes prevention programs, and beneficial effects exist when including behavioral support provided by a counselor [56]. Second, interventions targeted to the needs of specific communities is important to keep participants motivated. Therefore, participatory research (i.e. involving the end-user in each phase of the process, going from formative research to the evaluation of the intervention) seems to be an optimal method [57].

In several lifestyle behaviors within the current study, improvements from baseline to post-test in both the intervention and standard care group exist, which is a fourth factor that might have caused the limited observed behavioral changes intended by the intervention. These favorable evolutions in both groups might be explained by the fact that both the intervention and standard care group received feedback on families’ risk for developing type 2 diabetes, on parents’ blood indices and on parents’ and children’s BMI and step counts, possibly resulting in increased awareness among families.

As several studies showed clear differences in energy balance-related behaviors between European countries [9, 10, 58, 59] and inhabitants of different countries react in a different way on lifestyle intervention [60, 61], it is of great importance to tailor interventions to the needs of the target group on the one hand, and to local needs on the other hand. Therefore, within the current Feel4Diabetes-intervention, countries could make adaptations to counter for country specific needs or contextual circumstances. However, these efforts might be insufficient to reach the desired effects. Again, a bottom-up approach through conducting participatory research is recommended to optimal respond on the needs of young vulnerable families. Results of the current study revealed clear differences in significant intervention effects between the countries, which may have been caused by the local adaptations. For example, only in Belgium parenting practices regarding children’s lifestyle behaviors were addressed during counselling sessions and existing role modeling videos were used. This results in some favorable intervention effects of these parenting related-factors [62]. As parenting-related factors are a bridging function between the intervention and children’s health behaviors, improving some parenting-related factors may have caused an increase of Belgian children’s fruit and vegetable consumption.

Based on the results of this study, take home messages were formulated: (1) providing counseling sessions and mobile text messages are effective tools for improving certain lifestyle behaviors in families at risk for type 2 diabetes, however, more research is needed to find an optimal number of counseling sessions in order to conduct a cost-effective intervention; (2) keeping high attendance rates during the intervention remains a main challenge in this target group. Implementing e-health interventions or conducting participatory research (i.e. tailoring interventions) might respond on main perceived barriers such as lack of time or lack of motivation, possibly resulting in lower attrition rates and higher effectiveness; finally (3) providing intermediate feedback on families’ risk for type 2 diabetes and on results of measurements such as BMI, blood indices and step counts, will raise the awareness of families, possibly resulting in improvements on families’ lifestyle behaviors.

Strengths of the current study include the cluster-randomized design, the large European study sample which made it possible to investigate intervention effects across and within European countries, and the target on families at risk for developing type 2 diabetes. On the other hand, limitations exist in this study. The use of self-reported questionnaires to assess families’ lifestyle behaviors, potentially caused response bias. Second, country-specific analyses were conducted with < 150 families per treatment arm in all countries except from Greece. Based on a statistical power analysis, conducted before the start of the Feel4Diabetes-study, at least 150 families per treatment arm per country (intervention vs. control condition) were sufficient to reach the statistical power needed for this kind of intervention (> 80% power at a two-sided 5% significance level) to reduce BMI by 0.7 kg/m^2^ in adults within a year. Due to the large drop-out throughout the intervention, these figures (150 families per treatment arm per country) could not be reached after the first and second intervention year. Therefore, results should be interpreted with caution. Furthermore, for 60.4% of the Hungarian participants, researchers did unfortunately not indicate whether they were present or absent during the sessions. Third, findings can only be generalized to groups at risk for T2DM and living in low SES areas in Europe. Furthermore we believe that the generalizability of our findings deserves caution because of the high-drop-out figures. Finally, within the current study no adjustments were made for multiple testing, which may have increased the type 1 error rate.

## Conclusions

The Feel4Diabetes-intervention managed to improve a limited number of targeted lifestyle behaviors in families at risk for developing type 2 diabetes while the intervention was not effective on a large number of targeted lifestyle behaviors. The high attrition rate, observed especially in some countries, needs to be considered in future interventions in order to take corrective actions and improve their implementation. Further research is needed on how to develop and implement cost-effective lifestyle interventions to prevent type 2 diabetes in families at risk for developing type 2 diabetes.

## Supplementary Information

**Additional file 1. **Results of the longitudinal analyses on lifestyle behaviors in parents and children from families at risk for developing type 2 diabetes.

**Additional file 2.** CONSORT 2010 checklist of information to include when reporting a randomised trial*.

**Additional file 3.** The TIDieR (Template for Intervention Description and Replication) Checklist*.

## Data Availability

The data of the present study is available from the corresponding author on reasonable request.
