# Supplementary material for: The effect of a cluster-randomized controlled trial on lifestyle behaviors among families at risk for developing type 2 diabetes across Europe: the Feel4Diabetes-study
Source: Int J Behav Nutr Phys Act. 2021 Jul 1;18:86. doi: 10.1186/s12966-021-01153-4 (PMC8252328; doi:10.1186/s12966-021-01153-4)
Supplement: Supplementary file 1 — Additional file 1. Results of the longitudinal analyses on lifestyle behaviors in parents and children from families at risk for developing type 2 diabetes. [file 12966_2021_1153_MOESM1_ESM.docx]

Supplementary File 1: Results of the longitudinal analyses on lifestyle behaviors in parents and children from families at risk for developing type 2 diabetes.

| **Outcome variable** | **Country** | **F-value** | **P-value** |
| --- | --- | --- | --- |
| **Parents** | | | |
| **Water consumption** | **All countries** | **3.24** | **0.04** |
|  | Belgium | 6.28 | <0.01 |
|  | Finland | 0.09 | 0.91 |
|  | Greece | 2.65 | 0.07 |
|  | Hungary | 0.65 | 0.52 |
|  | Bulgaria | 0.22 | 0.81 |
|  | Spain | 0.04 | 0.96 |
| **Fruit and vegetable consumption** | All countries | 3.31 | 0.04 |
|  | Belgium | 1.60 | 0.20 |
|  | Finland | 0.94 | 0.39 |
|  | Greece | 1.59 | 0.21 |
|  | Hungary | 0.69 | 0.51 |
|  | Bulgaria | 1.45 | 0.24 |
|  | Spain | 1.84 | 0.16 |
| **Consumption of sweets** | **All countries** | **1.51** | **0.22** |
|  | Belgium | 0.78 | 0.46 |
|  | Finland | 2.09 | 0.13 |
|  | Greece | 0.85 | 0.43 |
|  | Hungary | 1.24 | 0.29 |
|  | Bulgaria | 2.79 | 0.06 |
|  | Spain | 3.66 | 0.03 |
| **Consumption of soft drinks and juices containing sugar** | **All countries** | **2.72** | **0.07** |
|  | Belgium | 0.67 | 0.51 |
|  | Finland | 0.43 | 0.65 |
|  | Greece | 0.64 | 0.53 |
|  | Hungary | 3.48 | 0.03 |
|  | Bulgaria | 0.54 | 0.59 |
|  | Spain | 0.15 | 0.86 |
| **Consumption of salty snacks and fastfood** | **All countries** | **0.40** | **0.67** |
|  | Belgium | 0.29 | 0.75 |
|  | Finland | 1.24 | 0.29 |
|  | Greece | 0.02 | 0.98 |
|  | Hungary | 0.10 | 0.91 |
|  | Bulgaria | 0.40 | 0.67 |
|  | Spain | 0.74 | 0.48 |
| **Breakfast consumption** | **All countries** | **1.24** | **0.29** |
|  | Belgium | 0.25 | 0.78 |
|  | Finland | 0.48 | 0.62 |
|  | Greece | 2.15 | 0.12 |
|  | Hungary | 1.70 | 0.19 |
|  | Bulgaria | 0.34 | 0.71 |
|  | Spain | 0.18 | 0.84 |
| **Physical activity** | **All countries** | **2.42** | **0.09** |
|  | Belgium | 3.87 | 0.02 |
|  | Finland | 0.51 | 0.60 |
|  | Greece | 0.21 | 0.81 |
|  | Hungary | 1.61 | 0.20 |
|  | Bulgaria | 0.72 | 0.49 |
|  | Spain | 1.35 | 0.26 |
| **Screen-time** | **All countries** | **1.28** | **0.28** |
|  | Belgium | 0.94 | 0.39 |
|  | Finland | 1.26 | 0.28 |
|  | Greece | 0.09 | 0.91 |
|  | Hungary | 0.13 | 0.88 |
|  | Bulgaria | 2.36 | 0.10 |
|  | Spain | 0.22 | 0.80 |
| **CHILDREN** | | | |
| **Consumption of water** | **All countries** | **0.28** | **0.75** |
|  | Belgium | 2.54 | 0.08 |
|  | Finland | 0.61 | 0.54 |
|  | Greece | 1.24 | 0.29 |
|  | Hungary | 0.48 | 0.62 |
|  | Bulgaria | 1.98 | 0.14 |
|  | Spain | 1.40 | 0.25 |
| **Fruit and vegetable consumption** | **All countries** | **1.44** | **0.24** |
|  | Belgium | 5.43 | <0.01 |
|  | Finland | 0.65 | 0.52 |
|  | Greece | 0.20 | 0.82 |
|  | Hungary | 0.33 | 0.72 |
|  | Bulgaria | 0.24 | 0.79 |
|  | Spain | 0.17 | 0.85 |
| **Consumption of sweets** | **All countries** | **5.13** | **<0.01** |
|  | Belgium | 0.26 | 0.78 |
|  | Finland | 2.49 | 0.08 |
|  | Greece | 1.43 | 0.24 |
|  | Hungary | 0.28 | 0.75 |
|  | Bulgaria | 0.12 | 0.89 |
|  | Spain | 5.42 | <0.01 |
| **Consumption of soft drinks and juices containing sugar** | **All countries** | **0.33** | **0.71** |
|  | Belgium | 0.91 | 0.41 |
|  | Finland | 1.27 | 0.28 |
|  | Greece | 1.80 | 0.17 |
|  | Hungary | 3.16 | 0.04 |
|  | Bulgaria | 0.16 | 0.86 |
|  | Spain | 2.24 | 0.11 |
| **Consumption of salty snacks and fastfood** | **All countries** | **2.89** | **0.06** |
|  | Belgium | 1.27 | 0.28 |
|  | Finland | 1.84 | 0.16 |
|  | Greece | 2.32 | 0.10 |
|  | Hungary | 1.19 | 0.31 |
|  | Bulgaria | 0.37 | 0.69 |
|  | Spain | INVALID | INVALID |
| **Breakfast consumption** | **All countries** | **0.75** | **0.47** |
|  | Belgium | 0.72 | 0.49 |
|  | Finland | 1.38 | 0.25 |
|  | Greece | 0.04 | 0.96 |
|  | Hungary | 0.19 | 0.83 |
|  | Bulgaria | 1.36 | 0.26 |
|  | Spain | 2.48 | 0.09 |
| **Physical activity** | **All countries** | **3.37** | **0.03** |
|  | Belgium | 1.02 | 0.36 |
|  | Finland | 4.52 | 0.01 |
|  | Greece | 1.23 | 0.29 |
|  | Hungary | 2.90 | 0.06 |
|  | Bulgaria | 0.77 | 0.46 |
|  | Spain | 0.01 | 0.99 |
| **Screen-time** | **All countries** | **0.95** | **0.39** |
|  | Belgium | 1.35 | 0.26 |
|  | Finland | 0.20 | 0.82 |
|  | Greece | 0.19 | 0.83 |
|  | Hungary | 0.84 | 0.43 |
|  | Bulgaria | 0.13 | 0.88 |
|  | Spain | 0.72 | 0.46 |

INVALID: No valid cases because of missing question
